# An Exploratory Study of Social Skills Deficits and Suicide Attempts in Adolescent Psychiatric Inpatients: A Machine Learning Analysis of Goldstein’s ART Framework

**DOI:** 10.3390/jcm15145436

**Published:** 2026-07-11

**Authors:** Przemysław Zakowicz, Monika Szewczuk-Bogusławska, Maksymilian A. Brzezicki, Tomasz Strawczyński, Maria Skibińska, Barbara Remberk

**Affiliations:** 1 Department of Neural Engineering and Space Medicine, Collegium Medicum, University of Zielona Góra, 65-417 Zielona Góra, Polandmariaski@ump.edu.pl (M.S.); 2 Centre for Child and Adolescent Treatment in Zabór, 66-003 Zielona Góra, Poland; t.strawczynski@cldim.zgora.pl; 3 Department of Psychiatry, Wroclaw Medical University, 50-367 Wroclaw, Poland; monika.szewczuk-boguslawska@umw.edu.pl; 4 Institute of Psychiatry and Neurology (IPiN), 50-367 Warsaw, Poland; bremberk@ipin.edu.pl

**Keywords:** suicide, adolescents, machine learning, social psychiatry

## Abstract

**Background/Objectives**: Social skills deficits are increasingly recognized as clinically relevant correlates of suicidal behavior in adolescents. Goldstein’s Aggression Replacement Training (ART) provides a structured framework for assessing discrete prosocial and emotional skills, but its relationship with suicidality in psychiatric inpatients has not been systematically examined. This paper investigated whether self-rated ART social skills items differentiate adolescents with and without a history of suicide attempt as well as whether machine learning (ML) models can identify interpersonal profiles associated with SA. **Methods**: In this cross-sectional study, 162 psychiatric inpatients aged 11–17 years (72% female) were assessed at admission. Social skills were measured using the 50-item Skillstreaming component of ART, which is grouped into six domains (basic, advanced, emotional, alternatives to aggression, stress management, planning). Machine learning models (logistic regression, random forest, XGBoost, SVM, k-NN, Gaussian Naive Bayes) were trained on item-level features using repeated nested cross-validation with a held-out test set providing an unbiased estimate of generalization. **Results**: One item—*Helping Others*—remained significant after Holm correction (adjusted *p* = 0.042) with higher scores among adolescents with a history of SA. Seven additional prosocial items reached nominal significance (raw *p* < 0.05) but did not survive correction. Across ML models, discrimination was modest (cross-validated ROC-AUC 0.59–0.66) with Gaussian Naive Bayes performing best (ROC-AUC 0.66). Held-out test set performance was lower (ROC-AUC 0.46, 95% CI 0.26–0.69). SHAP analysis localized the model’s signal to a coherent cluster of prosocial and empathic communication items with higher self-reported prosociality paradoxically associated with SA history. **Conclusions**: In this inpatient cohort, self-rated social skills carried a small but coherent discriminative signal for prior suicide attempt, which was concentrated in outward-directed prosocial behaviors rather than global interpersonal functioning. The counterintuitive directionality—higher prosocial self-ratings among attempters—aligns with developmental frameworks describing compliant, hyper-responsible, or self-silencing interpersonal styles. While predictive performance was modest and sample size limits generalizability, item-level ART assessment may generate hypotheses about interpersonal risk phenotypes in this specific population of predominantly female adolescent inpatients. Only one item survived correction for multiple comparisons, and all findings should be interpreted as exploratory and hypothesis generating.

## 1. Introduction

Suicide is one of the leading causes of premature mortality worldwide. The growing burden of suicide deaths and suicide attempts (SA) is one of the crucial public health problems among European countries particularly regarding the adolescent population. According to the World Health Organization, over 700,000 suicide deaths occur globally each year, and suicide is the fourth leading cause of death in the 15–29-year-old population. The epidemiology varies considerably depending on ethnicity, geographic region, and economic status [1]. Higher indexes of suicide were observed in middle-eastern European countries achieving over 10 per 100,000 deaths among male adolescents for Poland (2002) and Russia (2019), while significantly lower epidemiological burden is met in southern European countries (3–3.5/100,000) [2]. One of the most thoroughly described trends concerns sex differences with more than threefold higher mortality among males. Suicidal ideation is relatively common in the adolescent population and does not always lead to a suicide attempt or death, hence the need to better understand which factors differentiate adolescents who think about suicide from those who attempt it. At the same time, every suicide attempt is one of the most valuable predictors of a subsequent attempt and of suicidal death. On this basis, every instance of suicidal ideation and self-harm is conceptualized as a red flag in a mental-health crisis and should prompt multidisciplinary cooperation and personalized care.

According to recent data from the US Youth Risk Behavior Survey (2023), suicidal ideations (SI) confirmed over 20% of students with higher index for groups of minorities, like, e.g., non-heteronormative sexual orientation [3]. Regarding the suicide attempt, the authors noticed a 9.5% prevalence among adolescents with 13.3% for girls and 11.0% for boys [4]. The reporting of suicidal ideations is also one of the most frequent causes of emergency department admissions, reaching the value of 40 per 100,000 visits, which is generally and over twofold higher for adolescents [5].

Retrospective cohort studies on hospitalized adolescent populations indicate SI and SA as the main cause of psychiatric emergency care consultations with a significant impact of non-suicidal self-injuries (NSSI) in the risk ratio (OR near 4.0 for suicidal attempt). A large clinical analysis of over 1300 patients (2019–2023) on a Polish population revealed crucial psychosocial factors affecting the SA risk particularly for adolescent females, namely violence victimization, family conflicts, or educational problems [6]. Recent Polish emergency psychiatric consultations analysis underlined the role of school time (vs. winter holidays) as the precipitation factor [7]. These findings underline that hospitalized adolescents represent a population at exceptionally high risk, requiring a comprehensive assessment of interpersonal functioning and environmental factors. Suicidal epidemiology may reflect global crises like COVID-19 pandemics or war conflicts. Despite less consistent data from the different world areas [8], in Poland during the pandemics, an enormous increase was observed regarding SI and SA among adolescents; clinical observations confirmed the peak age for SA between 15 and 17 years old with self-poisoning as the most frequent method of SA [9]. The most confirmed risk factors of SA among adolescents, following the American Academy of Pediatrics, include the following: previous SA, NSSI, depression and anxiety disorders, substance misuse, bulling and peer violence and sexual/gender minority [10].

Recent research consistently showed that impairments in interpersonal functioning—including difficulties in peer relationships—are strongly associated with suicidal behaviors in adolescents. Meta-analyses and large cohort studies demonstrate that adolescents with a history of suicide attempt exhibit poorer peer relationship quality, greater social isolation, and higher levels of interpersonal distress compared with their non-suicidal peers [11,12]. Following the interpersonal theory of suicide (ITS), two crucial processes were identified as risk shaping: (i) thwarted belonginess and (ii) perceived burdensomeness [13]. Thwarted belonginess conceptualizes as a permanent feeling of social disconnection due to unmet need for personal bonds and relationships, the second term (ii) is defined as feeling of being a strain, harm, or cost on close relationships—leading to the perception that others would be better off without them. Studies show that adolescents with a history of suicide attempt demonstrate a reduced ability to build and maintain supportive relationships, which aligns with the predictions of ITS [13].

Despite the growing amount of research on adolescent suicidality, there are a lack of data on concrete social and behavioral mechanisms taking part in SA prediction models. One operationalized protocol for social functioning assessment is Goldstein’s Aggression Replacement Training (ART), which is widely used in clinical practice [14]. Although interpersonal difficulty is a well-replicated correlate of adolescent suicidality, most of this work treats social functioning globally, whereas social skills are in fact a set of separable competencies, and prosociality in particular is a multidimensional construct whose adaptiveness is not uniform [15]. Resolving discrete social skill items rather than a single composite therefore offers a way to ask which specific interpersonal competencies, if any, distinguish adolescent inpatients with and without a history of suicide attempt. In this paper, we aimed to track individuals’ self-assessment of social skills during ART protocol and the history of suicidal attempts of psychiatric adolescent inpatients. We used classification machine learning (ML) software differentiating particular social skills deficits as supportive for the retrospective risk of SA.

## 2. Materials and Methods

### 2.1. Participants and Protocol

This study was conducted with the approval of the local bioethics committee at Zielona Góra Chamber of Physicians as a part of broader scientific project (KEPLER study: 01/178/2025). All subjects were recruited from tertiary referral psychiatric center inpatients for western Poland (Children and Adolescent Treatment Centre in Zabór, Zielona Góra, Poland). The studied population encompassed n = 162 subjects (72% female), aged 15 ± 1 years. For 7 patients, we did not obtain suicidal data. Inclusion to the study was preceded by informed consent of the patient and/or the caregiver due to local law regulations. A clinical interview and retrospective data collection were performed at the admission to the center by a trained C&A psychiatry physician following both child/adolescent and caregiver information. The assessment of social competence following ART protocol was performed in the first week of hospitalization during occupational therapy (ART) by a trained C&A psychiatrist and psychotherapist. Participants were excluded if they presented with acute medical instability, including conditions requiring immediate intervention such as severe intoxication, loss of consciousness, respiratory compromise, or other life-threatening somatic emergencies. Individuals in an acute psychotic state or experiencing neurological crises, severe cognitive disorganization, or any condition preventing reliable assessment and informed participation were also excluded.

### 2.2. Social Skills Assessment: ART

Social skills were assessed using items derived from the Aggression Replacement Training (ART) model (Goldstein et al., 1998) [14], specifically the Skillstreaming component describing discrete prosocial competencies. The scale consists of 50 items, which are grouped into six blocks: **basic social skills** (e.g., initiating conversation, listening, asking questions), **advanced social skills** (negotiating, resolving conflicts, responding to peer pressure), **emotional skills** (recognizing and expressing emotions, empathic responding), **skills alternative to aggression** (managing impulses, constructive responses to provocation), **stress management skills** (identifying stress cues, applying calming strategies), and **planning skills** (setting goals, anticipating consequences, making decisions). Each item was self-rated individually by patient using a five-point Likert-scale reflecting the degree of competence (1 = almost never, 2 = rarely, 3 = sometimes, 4 = often, and 5 = almost always), enabling an item-level analysis of interpersonal and social competence.

### 2.3. Machine Learning Pipeline and Statistical Analysis

The candidate predictors were the 50 individual items of the social skills inventory, which are each scored from 1 to 5. We deliberately used the item-level scores rather than the six predefined domain averages, because items carry information that is collapsed when averaged and because including both items and their averages would have produced perfect collinearity; results using the parsimonious six-domain feature set were retained as a sensitivity analysis. Age was not included in the primary feature set in order to keep the model interpretable as a pure social-skills profile, but it is included in a sensitivity analysis (“items + age”). Item-level missing data were rare (one missing value in each of 13 of the 50 items, all other items complete) and were imputed using the training-fold median within the cross-validation pipeline so that no test-fold information could influence the imputation. We made no attempt to impute the outcome: participants without a usable suicide attempt count were excluded outright.

We did not perform a formal sample size calculation prior to analysis. The dataset comprises the available consecutive inpatient sample at this service, and we report it as such. We acknowledge that 162 participants with 50 candidate predictors lies well below the events-per-variable thresholds suggested for stable prediction modeling [16], and we frame all results accordingly as exploratory and hypothesis-generating rather than as a finalized model ready for deployment. This study is reported in accordance with the TRIPOD+AI statement [17]; a completed checklist is provided in the Supplementary Material.

### 2.4. Modeling Pipeline and Validation

Before any modeling, we partitioned the 155 participants into a development set of 124 and a held-out test set of 31, using a single stratified split that preserved the outcome prevalence in both partitions (suicide attempt prevalence 42.7% in development, 41.9% in test). The held-out test set was set aside and accessed exactly once, after the entire model selection process was complete, in order to provide an honest estimate of out-of-sample performance that was not contaminated by repeated looks. All decisions about preprocessing, imbalance correction, candidate model choice, and hyperparameter selection were made on the development set alone.

Within the development set, we estimated model performance using repeated nested cross-validation with five outer folds, five inner folds, and five repeats, giving 25 outer evaluations per candidate model. We chose nested rather than single-loop cross-validation because performance estimates obtained from the same cross-validation loop used to tune hyperparameters are upwardly biased, which is an effect that is particularly pronounced at small sample sizes [18,19]. All preprocessing steps, namely median imputation and standardization to zero mean and unit variance, were fitted on the inner training folds only and applied to the inner validation folds, eliminating preprocessing leakage. Imbalance handling was governed by an a priori rule: class weighting if the minority share lay between 10% and 40%, SMOTE oversampling within training folds [20] if the minority share fell below 10%, and no correction otherwise. With an observed minority share of 42.7%, the rule yielded no correction. The decision rule was specified in advance to avoid any post hoc selection of an imbalance strategy on the basis of cross-validated performance.

We compared six candidate models that span the linear-to-non-linear and parametric-to-non-parametric spectrum: penalized logistic regression (L2), random forest, gradient boosted trees (XGBoost), support vector machine with a radial basis function kernel, k-nearest neighbors, and Gaussian Naive Bayes. A stratified-prediction dummy classifier was included as a sanity-check baseline. For each candidate, hyperparameters were tuned by a 30-iteration randomized search over reasonable prior ranges (penalty strength for logistic regression and SVM; tree depth, number of estimators, and learning rate for the tree ensembles; neighbor count for k-NN; variance smoothing for Naive Bayes) with selection driven by the inner-fold area under the receiver operating characteristic curve (ROC-AUC). On each outer fold, we recorded ROC-AUC as the primary discrimination metric, average precision (PR-AUC) as a class-imbalance-robust complement, the Brier score as a measure of overall fit and calibration, and balanced accuracy and the Matthews correlation coefficient as threshold-dependent class-balanced summaries. The model with the highest mean outer-fold ROC-AUC across the 25 evaluations was selected as the final model.

The selected model was then refit on the entire development set with hyperparameters re-tuned by inner cross-validation, and then it was applied to the held-out test set. Test set performance is reported with 95% confidence intervals derived from 1000 bootstrap resamples of the test predictions; we used the percentile method, which makes no parametric assumption about the sampling distribution of the metrics. Univariate associations between each item and the outcome were tested on the full sample using the Mann–Whitney U test with effect sizes reported as the rank–biserial correlation; *p*-values were adjusted for multiple comparisons across the 50 items by the Holm step-down procedure. These univariate analyses were exploratory and were not used for feature selection in the modeling pipeline.

## 3. Results

### 3.1. Clinical and Demographic Data of Studied Population

The studied population included 162 adolescents (72% female) aged 11–17 years (M: 15, SD: ±1) with the most frequent diagnoses being conduct and emotional disorder (F91–93, following ICD-10 code): n = 78 (48%), major depressive disorder: n = 27 (17%) and anxiety-stress related disorders (F4x following ICD-10 code), n = 8 (5%). Detailed demographic and clinical data are presented in the table below (Table 1). For 39 patients (24%), psychoactive substances misuse was reported in the medical history with the most frequent substances being alcohol, nicotine, cannabis and psychostimulants. Out of 162 adolescents, 69 (43%) were hospitalized for the first time (M: 2, SD: ±1.8, Max. 10 times). For 100 (62%) of the adolescents, non-suicidal self-injuries (NSSI) were reported, 72 patients (44%) had undertaken a suicide attempt (SA) using a violent method (n = 31, 43% of SA) or non-violent method (n = 41, 57%), and the most frequent suicide attempt method was drug self-poisoning (n = 33, 46% of SA). The mean number of SA was 1 (SD: ±1.5, Max.: 10).

### 3.2. Social Competence: Descriptive Analysis

Univariate testing across the 50 inventory items identified one item that survived Holm correction at the conventional 5% significance threshold. Higher self-reported scores on item 24, “Helping Others,” were associated with a history of suicide attempt (Mann–Whitney U = 2052, raw *p* = 0.00084, Holm-adjusted *p* = 0.042) with a rank-biserial effect size of 0.29 indicating a small-to-moderate effect. A further seven items reached nominal significance at raw *p* < 0.05 but did not survive correction for multiple testing: “Giving a Compliment,” “Understanding the Feelings of Others,” “Introducing Other People,” “Being a Good Sport,” “Following Instructions,” “Apologizing,” and “Keeping Out of Fights” with rank-biserial effect sizes ranging from 0.18 to 0.26. All eight items pointed in the same direction, with consistently higher self-reported social skills scores in participants with a history of suicide attempt, all of which were clustered within the prosocial and empathic communication subdomains of the inventory rather than being scattered across the full instrument.

**Figure 1:**
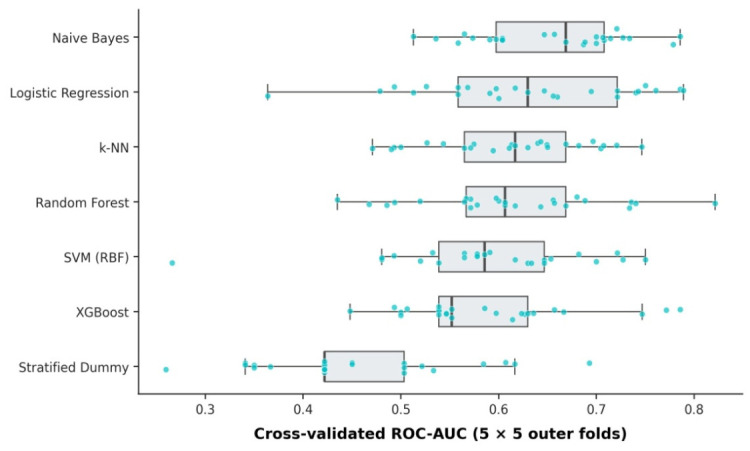
Per-outer-fold ROC-AUC for each candidate model and the stratified-prediction baseline from repeated nested cross-validation (5 outer folds × 5 repeats = 25 outer evaluations). Boxes show the interquartile range and median; individual points show the 25 outer-fold scores per model.

Repeated nested cross-validation across the six candidate models and the stratified baseline produced the performance summary shown in Table 2 and visualized by outer-fold distribution in Figure 1. All six real models outperformed the stratified prediction baseline (mean ROC-AUC 0.45), confirming that the social skills features carried discriminative information beyond what could be obtained from the marginal class distribution alone. Performance was, however, modest in absolute terms and clustered tightly across the six models with mean ROC-AUC ranging from 0.59 to 0.66 and overlapping standard deviations across outer folds. Gaussian Naive Bayes achieved the highest mean ROC-AUC (0.66, SD 0.07), which was followed by penalized logistic regression (0.63, SD 0.11). The two tree-based ensembles, random forest and XGBoost, did not improve on the linear baselines and in fact performed marginally worse, which is a pattern that is consistent with the limited sample size and the absence of strong non-linear interactions in this dataset. The Matthews correlation coefficient told the same story: positive but small for all real models (0.07 to 0.19) and clearly negative for the stratified dummy. On the basis of the highest mean primary metric, Naive Bayes was selected as the final model. The selected hyperparameter, a variance-smoothing constant of 1.8 × 10^−10^, lay near the lower end of the search range, indicating that the model relied on the empirical class-conditional variances of the features without strong regularization.

When the refit Naive Bayes model was applied for the first and only time to the held-out test set of 31 participants (of whom 13 had a documented suicide attempt), point-estimate discrimination was lower than the cross-validation estimate (test set ROC-AUC 0.46, 95% bootstrap CI 0.26 to 0.69) and the test set Brier score was 0.40, indicating that the model’s predicted probabilities were poorly calibrated to the observed event rate at this sample size. At the default 0.5 decision threshold, the model achieved a sensitivity of 0.69 (9 of 13 attempt cases correctly identified) at the cost of a precision of 0.49 (9 false positives among 18 negative cases), giving a balanced accuracy score of 0.59 and a macro-F1 value of 0.57. Two observations are important for interpretation. First, every test-set bootstrap confidence interval was wide and the ROC-AUC interval crosses 0.5, so the test-set point estimate is consistent with both clinically meaningful discrimination and chance-level performance. Second, the gap between the cross-validation estimate (ROC-AUC 0.66) and the held-out estimate (0.46) is large, but a single stratified test-set draw of 31 participants is itself a high-variance estimator of generalization performance, and the cross-validation estimate, derived from 25 outer evaluations on 124 patients, is the more stable summary at this sample size (Vabalas et al., 2019 [19]). We therefore report the cross-validation ROC-AUC of 0.66 as the primary performance estimate and the held-out ROC-AUC of 0.46 as a confirmatory check while noting the divergence as a finding in its own right.

To address whether the choice of Gaussian Naive Bayes, which assumes conditional independence of the 50 correlated items, drove these results, we also refit penalized logistic regression on the full development set and applied it once to the held-out test set. Discrimination was indistinguishable between the two models (logistic regression test ROC-AUC 0.50, 95% bootstrap CI 0.31 to 0.71; Naive Bayes 0.46, 95% CI 0.26 to 0.69), but logistic regression was substantially better calibrated (Brier 0.24 versus 0.40), which is consistent with the expectation that the Naive Bayes calibration failure reflects its violated independence assumption. At the default 0.5 threshold, the better-calibrated linear model, which was shrunk toward the base rate, classified almost all held-out cases as non-attempters, whereas Naive Bayes achieved nominal sensitivity at the cost of calibration. Neither test-set confidence interval excluded 0.5. At this sample size, the choice among algorithms is therefore not identifiable, and the two leading models agree on the location and direction of the signal while differing in calibration in the manner that the theory predicts. Because the model is used here only to localize where the univariate signal sits in the instrument, and not as a deployable predictor, the feature rankings derived from it should be read as descriptive and unstable at this events-per-variable ratio.

To test whether the cross-validated discrimination could be an artifact of fitting 50 features to a small development sample, we re-estimated the development-set cross-validated ROC-AUC under 300 random permutations of the outcome labels, holding the pipeline fixed. The permutation null distribution was centered at chance (mean 0.50, SD 0.07, 95th percentile 0.60), and the observed value of 0.66 fell in the upper tail (one-sided permutation *p* = 0.01). The in-sample signal is therefore unlikely to be a chance product of the feature-to-sample ratio, even though, as the held-out results show, it does not generalize robustly at this sample size.

To test whether the single Holm-corrected association was independent of major clinical predictors, we modeled item 24, Helping Others, in a logistic regression adjusted for sex, NSSI, psychoactive-substance use, conduct or emotional disorder, and depressive disorder. The association remained significant after adjustment (unadjusted odds ratio per one-point increase 2.06, 95% CI 1.36 to 3.12; fully adjusted odds ratio 1.79, 95% CI 1.12 to 2.86, *p* = 0.016). Female sex was, as expected, independently associated with attempt history (odds ratio 2.96, 95% CI 1.17 to 7.49), as was NSSI (odds ratio 2.46, 95% CI 1.08 to 5.63); adjustment for sex modestly attenuated but did not eliminate the prosocial signal. The number of hospitalizations was not included as a covariate, as it is plausibly a consequence of prior attempts rather than a confounder. These item-level comparisons were performed on the 155 participants with a usable outcome (66 with a history of attempt, 89 without; sex was available for all 162 participants).

The internal consistency of the six ART domains in this sample, estimated by Cronbach’s alpha, ranged from acceptable to good—Beginning Social Skills 0.71, Advanced Social Skills 0.56, Dealing with Feelings 0.64, Alternatives to Aggression 0.67, Dealing with Stress 0.79, and Planning 0.86—with an alpha of 0.93 for the full 50-item scale. The Advanced Social Skills domain, with the fewest items and the lowest alpha, should be interpreted with caution. These are internal-consistency estimates only and are not a substitute for formal validation of the Skillstreaming inventory as a psychometric scale in adolescent inpatients.

**Figure 2:**
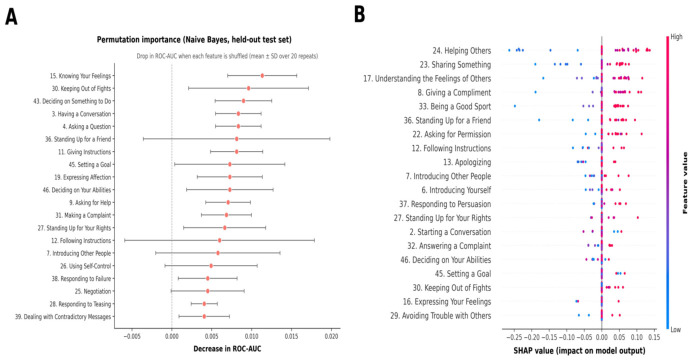
Two complementary views of feature importance for the final Naive Bayes model on the held-out test set. (**A**) Permutation importance: mean drop in test-set ROC-AUC (±SD over 20 shuffling repeats) when each feature is randomly permuted; the 20 features with the largest mean drop are shown. (**B**) SHAP summary plot: each point is one test-set participant, the horizontal position shows the SHAP contribution of that feature to the model’s log-odds output for that case, and the color shows the participant’s score on that feature (blue = low, red = high). Features in panel B are ordered by mean absolute SHAP value with the most influential at the top. Positive SHAP values shift the prediction toward the suicide-attempt class. *In clinical terms, the leading items can be read directly: higher self-rated competence on “Helping Others,” “Sharing Something,” “Understanding the Feelings of Others,” and “Giving a Compliment”—that is, an adolescent who rates themselves as markedly more helpful, empathic, and accommodating than peers—pushed the model toward the prior-attempt class, whereas lower self-rated help-seeking and self-directed skill pushed it the other way. The plot therefore localizes the signal to outward-directed prosocial behaviors rather than to global social competence*.

Because aggregate discrimination metrics tell the clinician nothing about which features the model relies on or in which direction they act, we examined the explainability of the final model using two complementary lenses (Figure 2). Permutation importance on the held-out test set (Figure 2A) identified “Knowing Your Feelings” (item 15) as the feature whose shuffling produced the largest mean drop in ROC-AUC, which was followed by “Keeping Out of Fights” (item 30), “Deciding on Something to Do” (item 43), “Having a Conversation” (item 3), and “Asking a Question” (item 4). All top-ranked permutation importances were small in absolute terms (mean AUC drops of 0.005 to 0.012), and the standard deviations across the 20 shuffling repeats overlapped substantially across the top-20 list, indicating that the deployed model does not rely heavily on any single feature for held-out discrimination. SHAP analysis on the same test set (Figure 2B) produced a complementary but partially divergent picture. The single dominant SHAP feature was item 24, “Helping Others,” with a mean absolute SHAP value of 0.094, which was more than twice that of any other feature; it was followed by “Sharing Something” (0.046), “Understanding the Feelings of Others” (0.040), “Giving a Compliment” (0.037), “Being a Good Sport” (0.031), and “Standing Up for a Friend” (0.030). The SHAP ranking aligned closely with the univariate testing: every feature that reached nominal univariate significance appeared in the SHAP top 10. The two views are consistent rather than contradictory: SHAP measures each feature’s average marginal contribution to the model’s log-odds output across cases, while permutation importance measures how much the model needs that feature to maintain rank-order discrimination on held-out data. A feature can carry strong individual-prediction weight (high SHAP) and yet, because Naive Bayes treats features as conditionally independent, its loss can be partially compensated by correlated items when the column is shuffled (modest permutation importance). Critically, the directionality visible in Figure 2B confirms the univariate finding at the model level: across the top-ranked SHAP features, higher feature values (red points to the right of zero) pushed the prediction toward the suicide-attempt class, while lower feature values pushed it toward the no-attempt class. Taken together, the two explainability views localize the model’s discriminative signal, modest as it is in aggregate, to a coherent subset of prosocial and empathic communication items rather than to the inventory as a whole, and they identify the same direction of effect: in this inpatient cohort, higher self-reported prosocial skills is the marker of, rather than protection against, prior suicide attempt.

## 4. Discussion

In this exploratory study of adolescent psychiatric inpatients, self-rated social skills carried a modest discriminative signal for prior suicide attempt. The signal was concentrated in a coherent cluster of outward-directed prosocial items, on which attempters self-rated higher than non-attempters, while ratings on help-seeking and self-directed items showed the opposite trend. Out-of-sample performance was unstable, as expected given the sample size, and no algorithm in our comparison materially outperformed penalized logistic regression.

Our discriminative ceiling is consistent with the broader empirical landscape. Single risk factors predict suicidal thoughts and behaviors at near-chance levels even when measured prospectively [21], and the area under the curve for machine learning models of adolescent suicide attempt reaches its higher values only in electronic-health-record cohorts with thousands of patients and hundreds of features [22]. A single-modality self-report instrument in 155 inpatients is not the regime in which clinically deployable prediction is plausible [16], and we present our model accordingly.

The directionality of the social skills signal is more interesting than its magnitude. The interpersonal theory of suicide would predict that adolescents with a history of attempt should self-report poorer interpersonal functioning [13,14,16,17,18,19,20,21,22,23], and several adolescent inpatient studies report exactly this [24,25]. We found the opposite, and the pattern was content-specific rather than global: attempters self-rated as more helpful, more empathic, more compliant and more conflict-avoidant, but as less able to ask for help, prepare for a difficult conversation, or reward themselves. A uniform positive self-presentation bias would have lifted every item, and it did not.

We advance three convergent frameworks as candidate hypotheses for this configuration, recognizing that the present cross-sectional, self-report design cannot establish any of them over the alternatives considered in the limitations. In the object-relations tradition, Winnicott’s false self develops when the early caregiving environment is insufficiently responsive to the child’s spontaneous gestures; the resulting compliant, caregiver-pleasing surface is built to anticipate and meet the needs of others, and it is structurally incompatible with letting itself be cared for [26,27]. The clinical phenotype of parentification describes a closely related developmental position in which a child takes on age-inappropriate emotional caregiving and presents as hyper-responsible, attuned, and self-silencing with elevated risk of internalizing symptoms and self-harm [28]. In the sociological literature, Abrutyn and Mueller’s revival of the Durkheimian altruistic type proposes that hyper-integration into a tightly-knit relational system, rather than disconnection from it, can generate suicidal vulnerability when relational worth becomes contingent on what is given to others and when help seeking is read as a breach of integration [29,30,31]. Family-systems theory offers the proximate developmental mechanism through the identified-patient construct, in which the most relationally accommodating member of a dysfunctional family system carries the system’s affective load and discharges it through self-harm. Across these frameworks, the high-prosocial, low-help-seeking profile we observed is consistent with a recognized developmental phenotype rather than necessarily reflecting a measurement artifact—a phenotype that, if genuine, would map onto the perceived-burdensomeness construct at the level of behavior rather than belief. The clinical implication is that ceiling-level self-rated prosocial competence on a face-valid instrument in an inpatient adolescent should be interrogated rather than reassured.

This pattern is best read against a multidimensional understanding of prosociality. Prosocial behavior is not a single disposition but rather a composite of helping, sharing, and comforting tendencies that draw on partly separable socio-cognitive, empathic, evaluative, and action-planning processes that do not necessarily covary [15]. Within that composite, the adaptiveness of prosociality has limits. Recent work validating a measure of compulsive helping, defined as helping that harms the helper, found it positively associated with anxiety and negatively associated with self-regulation, which suggests that high outward-directed helping can index distress rather than resilience [32]. Two further considerations bear directly on our finding. First, because the ART is a self-report instrument, it captures perceived prosocial competence, which need not correspond to observable prosocial behavior; the discrepancy between perceived and enacted prosociality is exactly where a compliant or self-presentational style would be expected to inflate scores. Second, whether prosociality protects or harms appears to depend on its motivational and emotional substrate: autonomously motivated helping is associated with greater well-being for the helper [33], whereas empathically over-engaged caregiving, the “cost of caring,” is associated with internalizing distress and is more pronounced in girls [34], which is a sex difference consistent with the female predominance of our cohort. These studies in the literature suggest that empathy, emotional intelligence, social support, resilience, and relationship quality are plausible moderators of the direction of effect we observed, and the same self-rated prosocial profile may be protective in one configuration and a marker of vulnerability in another. The present cross-sectional design cannot test these mediating pathways, and we advance them as directions for multimodal replication rather than as established mechanisms.

One should also bear in mind that the ART is a self-assessment scale. Therefore, self-reported prosocial emotions may not necessarily reflect an individual’s actual capacity to build satisfying relationships. For example, prosocial emotions accompanied by inadequate communication may occur in participants with autism spectrum disorder. Similarly, prosocial declarations that are not followed by prosocial behaviors may constitute part of an antisocial functioning pattern. Thus, declared prosocial emotions may reflect a longing for peer friendship—which is itself consistent with known suicide risk factors—rather than the presence of genuinely supportive peer relationships.

This paper has several limitations. The cohort is small, single-center, and predominantly female, which constrains generalizability. The design is cross-sectional and the outcome was ascertained retrospectively, so the social-skills profile cannot be positioned causally with respect to the attempt. All exposures are self-reported in an inpatient context, with no observer-rated or behavioral anchor, and the feature-to-event ratio in the development set is well below thresholds for stable prediction modeling [16]. Diagnostic comorbidity, NSSI, substance use and medication were deliberately excluded to preserve interpretation as a pure social-skills profile, which means the model is not a competitor to clinical risk stratification. Replication in independent inpatient cohorts using observer-rated or behavioral measures alongside self-report, with explicit modelling of social-desirability bias, would establish whether the prosocial direction-of-effect we report is reproducible. If it is, it would refine the perceived-burdensomeness construct by identifying a hyper-caregiving, help-suppressing phenotype as a distinct adolescent attempt subtype with implications for assessment and for the targets of psychological intervention.

Several further limitations qualify the interpretation. Because exposure was measured in the first week of hospitalization, after admission and after the patient’s clinical status was known to them, the temporal ordering of the social-skills self-report and the attempt cannot support a causal or directional reading, and differential retrospective reporting between attempters and non-attempters cannot be excluded. The counter-theoretical direction of the prosocial signal is consistent with several explanations that the present design cannot distinguish: a genuine high-prosociality, low-help-seeking phenotype; social-desirability or impression-management bias elevated in inpatients; mood-congruent positive self-appraisal; and response acquiescence differing between groups. We regard a uniform response-style account as less likely because the observed pattern was content-specific rather than a global upward shift with attempters rating outward-directed prosocial items higher but self-directed and help-seeking items lower, but we cannot exclude it because no social-desirability scale or observer rating was administered. The female predominance means the findings apply chiefly to female adolescent inpatients and may not transfer to mixed-sex or community samples; although the corrected association survived adjustment for sex, the small number of male participants (n = 45) limits the precision of any sex-stratified inference. The diagnostic composition, dominated by conduct and emotional disorder, differs from the populations in which the interpersonal, parentification, and false-self frameworks were developed, so those frameworks are imported here as candidate explanations to be tested rather than as validated descriptions of this cohort. Self-perception bias and the absence of hetero-rated measures should therefore be borne in mind throughout.

## 5. Conclusions

In a single-center cohort of adolescent psychiatric inpatients, item-level self-rated pro-social declarations carried a modest, exploratory signal for prior suicide attempt that did not generalize robustly to a held-out subset. The signal was directional rather than diffuse: attempters self-rated as more prosocial and conflict-avoidant but less able to seek help or accept comfort, which is a profile that is consistent with the false-self, parentification and altruistic-Durkheimian phenotypes long described in the clinical and sociological literature. Read in this light, our findings refine rather than contradict the interpersonal theory of suicide, and they argue against treating high self-rated prosocial competence in acute inpatient adolescents as a marker of resilience. Larger, multimodal, multi-site replication with observer-rated anchors is required before any clinical inference is drawn.

## Figures and Tables

**Table 1:** (**a**) Clinical and demographic data of studied population. (**b**) Clinical and diagnostic profile by suicide attempt status for the 155 participants with a documented suicide attempt outcome. Group comparisons for binary variables by Chi-square test.

(**a**)					
	**General (n = 162)**	**Females (n = 116)**		**Males (n = 46)**	***p* (M vs. F)**
Age (M ± SD)	15 (±1)	15 (±1.5)		15(±0.2)	0.88 ^2^
NSSI	100 (62%)	82 (71%)		18 (39%)	**<0.05** ^1^
SA	72 (44%)	64 (55%)		8 (17%)	**<0.05** ^1^
Number of SA (M ± SD)	1 (±1.5)	1 (±1.7)		0 (±0.6)	**<0.05** ^2^
Number of hospitalizations (M ± SD)	2 (±1.8)	2 (±1.8)		2 (±1.6)	0.78 ^2^
Psychoactive substances use	39 (24%)	27 (23%)		12 (26%)	0.71 ^1^
(**b**)					
	**Attempters (n = 66)**		**Non-attempters (n = 89)**		***p***
Female, n (%)	57 (86%)		53 (60%)		<0.001 ^1^
NSSI, n (%)	53 (80%)		45 (51%)		<0.001 ^1^
Psychoactive substance use, n (%)	23 (35%)		24 (27%)		0.38 ^1^
Number of hospitalizations (M ± SD)	2.7 (±2.0)		2.1 (±1.6)		—
Primary diagnosis, n					
Conduct/emotional (F90–98)	31		35		
Depressive (F32–33)	14		11		
Anxiety/stress-related (F40–48)	8		10		
Neurodevelopmental (F80–89)	3		12		
Psychotic (F20–29)	0		5		
Personality (F60–69)	2		3		
Other/unspecified	8		13		

(**a**) ^1^ Chi-square, ^2^ *t*-test. (**b**) ^1^ Chi-square test. The full sample comprises 162 adolescents; 7 had missing suicide attempt data and were excluded from outcome analyses, leaving 155 (66 with and 89 without a history of attempt). Counts in the diagnosis block sum to the group totals.

**Table 2:** Discrimination, calibration, and class-balanced performance of candidate models estimated by repeated nested cross-validation on the development set (5 outer × 5 inner folds, 5 repeats, 25 outer evaluations per model). Values are mean (standard deviation) across outer folds. ROC-AUC, area under the receiver operating characteristic curve; PR-AUC, area under the precision–recall curve; MCC, Matthews correlation coefficient.

Model	ROC-AUC	PR-AUC	Brier	Balanced acc.	MCC
Naive Bayes	0.66 (0.07)	0.61	0.31	0.59 (0.07)	0.19 (0.14)
Logistic regression	0.63 (0.11)	0.58	0.25	0.57 (0.08)	0.15 (0.19)
k-Nearest neighbors	0.61 (0.08)	0.54	0.24	0.55 (0.07)	0.10 (0.16)
Random forest	0.61 (0.09)	0.55	0.24	0.55 (0.06)	0.12 (0.14)
SVM (RBF)	0.59 (0.10)	0.54	0.24	0.54 (0.06)	0.07 (0.11)
XGBoost	0.59 (0.09)	0.53	0.26	0.55 (0.08)	0.10 (0.16)
Stratified baseline	0.45 (0.10)	0.42	0.53	0.45 (0.10)	−0.10 (0.20)

Held-out test-set performance (n = 31) for the two leading models evaluated once after model selection: Gaussian Naive Bayes ROC-AUC 0.46 (95% CI 0.26–0.69), Brier 0.40; penalized logistic regression ROC-AUC 0.50 (95% CI 0.31–0.71), Brier 0.24. Both confidence intervals include 0.5; logistic regression, which does not assume feature independence, was better calibrated.

## Data Availability

The anonymized dataset supporting the findings of this paper is available from the corresponding author upon reasonable request. Although all data have been fully anonymized, they contain sensitive clinical information and therefore cannot be made publicly accessible.

## Supplementary Materials

The following supporting information can be downloaded at https://www.mdpi.com/article/10.3390/jcm15145436/s1.

## Abbreviations

The following abbreviations are used in this manuscript:

ART	Aggression Replacement Training
SA	Suicide attempt
NSSI	Non-suicidal self-injury
ML	Machine learning
