# Supplementary material for: An Exploratory Study of Social Skills Deficits and Suicide Attempts in Adolescent Psychiatric Inpatients: A Machine Learning Analysis of Goldstein’s ART Framework
Source: J Clin Med. 2026 Jul 11;15(14):5436. doi: 10.3390/jcm15145436 (PMC13412294; doi:10.3390/jcm15145436)
Supplement: Supplementary file 1 [file jcm-15-05436-s001.zip › jcm-4359960-supplementary.pdf]

Supplementary material: TRIPOD+AI reporting checklist

Social Skills Deficits and Suicide Attempts in Adolescent Psychiatric Inpatients: A Machine Learning Analysis of Goldstein's ART Framework

Completed against the 27-item TRIPOD+AI checklist (Collins GS, Moons KGM, Dhiman P, et al. BMJ. 2024;385:e078378. doi:10.1136/bmj-2023-078378). "Done" records whether the item is addressed; "Partial" and "N/A" are explained in the comment. As an exploratory single-cohort study with internal validation only, items relating to external validation, clustering, and model updating are not applicable.

Item	Checklist item	Done	Location / comment
**Title and abstract**			
1	Title: identify study as developing or evaluating a multivariable prediction model, target population, outcome.	Yes	Title ("Machine Learning Analysis"; adolescent psychiatric inpatients; suicide attempts).
2	Abstract per TRIPOD+AI for Abstracts checklist (design, data, outcome, discrimination and calibration with CIs).	Yes	Structured abstract reports ROC-AUC and Brier with 95% CI and the single corrected item.
**Introduction**			
3a	Healthcare context and rationale; whether diagnostic or prognostic; references to existing models.	Yes	Introduction, paras 1–5 (interpersonal theory; absence of social-skills prediction models).
3b	Target population and intended purpose in the care pathway; intended users.	Partial	Introduction final para and Discussion: exploratory phenotype characterisation, not a deployable tool.
3c	Known health inequalities between sociodemographic groups.	Yes	Introduction (sex differences, minority status); Discussion/Limitations (female predominance).
4	Study objectives; development, evaluation, or both.	Yes	End of Introduction: develops and internally evaluates an exploratory model.
**Methods**			
5a	Sources of data for development and evaluation; rationale; representativeness.	Yes	Methods 2.1; single-site consecutive inpatient sample; representativeness discussed in Limitations.
5b	Dates of participant accrual; end of follow-up if applicable.	Partial	Cross-sectional; assessment at admission, first week of hospitalisation (Methods 2.1).
6a	Study setting; number and location of centres.	Yes	Methods 2.1: single tertiary referral centre, Zabór, western Poland.
6b	Eligibility criteria.	Yes	Methods 2.1: inclusion/exclusion (acute instability, psychosis, cognitive disorganisation).
6c	Treatments received and how handled, if relevant.	N/A	Not a treatment-effect study; clinical variables deliberately excluded from the model (Methods 2.3).
7	Data pre-processing and quality checking; consistency across groups.	Yes	Methods 2.3–2.4: median imputation and standardisation within CV folds.
8a	Outcome definition, time horizon, how/when assessed, rationale.	Yes	Methods 2.1: history of suicide attempt by clinical interview (patient and caregiver) at admission.
8b	Qualifications/characteristics of outcome assessors (if subjective).	Yes	Methods 2.1: trained child and adolescent psychiatrist.
8c	Actions to blind outcome assessment.	Partial	Limitations: outcome ascertained at admission; temporal/contextual confounding acknowledged.
9a	Choice of initial predictors and any pre-selection.	Yes	Methods 2.3: all 50 item-level scores used; no univariate pre-selection for the model.
9b	Definition of all predictors; how/when measured.	Yes	Methods 2.2: 50 Skillstreaming items, 5-point Likert, self-rated in first week.
9c	Qualifications/characteristics of predictor assessors (if subjective).	Yes	Methods 2.2: self-report administered during occupational therapy by trained staff.

Item	Checklist item	Done	Location / comment
10	How study size was arrived at; justification of sufficiency; sample size calculation.	Yes	Methods 2.3: no formal calculation; consecutive sample; EPV limitation stated explicitly (ref Riley).
11	Handling of missing data; reasons for omitting data; imputation separate for train/test.	Yes	Methods 2.3: ≤1 missing value per item; fold-internal median imputation; 7 missing outcomes excluded.
12a	How data were used; partitioning; sample size considerations; no leakage.	Yes	Methods 2.4: single stratified 124/31 split; held-out set accessed once.
12b	Handling of predictors (functional form, rescaling, standardisation).	Yes	Methods 2.4: standardisation to zero mean and unit variance within folds.
12c	Model type, rationale, model-building steps, hyperparameter tuning, internal validation.	Yes	Methods 2.4: six models; repeated nested CV (5×5×5); randomised hyperparameter search.
12d	Heterogeneity across clusters.	N/A	Single centre; no clustering.
12e	Measures and plots to evaluate performance; comparison of multiple models.	Yes	Methods 2.4: ROC-AUC, PR-AUC, Brier, balanced accuracy, MCC; model comparison by mean CV ROC-AUC.
12f	Model updating arising from evaluation.	N/A	No recalibration or updating performed.
12g	How model predictions are calculated (for evaluation).	Yes	Methods 2.4 and Results 3.2; held-out predictions from refit pipeline; code available on request.
13	Class imbalance methods; rationale; recalibration.	Yes	Methods 2.4: a priori rule; observed minority share 42.7%, no correction applied.
14	Approaches to model fairness and rationale.	Partial	Limitations: female predominance and single-site composition limit fairness/generalisability; sex not coded.
15	Model output (probabilities, classification); thresholds.	Yes	Results 3.2: probability output; default 0.5 threshold reported with sensitivity/precision.
**Results**			
16	Flow of participants; numbers at each stage; reasons for exclusion.	Yes	Results 3.1; Methods 2.4: 162 total, 7 excluded for missing outcome, 155 analysed (124 dev / 31 test).
17	Participant characteristics; outcome distribution; by-group comparison.	Yes	Results 3.1; Table 1 and new Table 1b (by attempter status, including diagnosis, NSSI, substance use).
18	Model specification and performance (development).	Yes	Results 3.2; Table 2: nested-CV discrimination, calibration, class-balanced metrics for six models.
19	Model performance on evaluation/held-out data with uncertainty.	Yes	Results 3.2 and Table 2 footnote: held-out ROC-AUC with bootstrap 95% CI for NB and logistic regression.
20	Model interpretability / explainability where applicable.	Yes	Results 3.2; Figure 2: SHAP and permutation importance, with stability caveat; permutation-null test added.
**Discussion**			
21	Overall interpretation; fairness; comparison with prior work.	Yes	Discussion paras 1–4; single corrected finding framed against the interpersonal theory and prosociality literature.
22	Limitations (non-representative sample, sample size, overfitting, missing data) and effects on bias and generalisability.	Yes	Limitations: small single-site female-predominant cohort, EPV, cross-sectional retrospective outcome, self-report, no sex variable.
**Other information**			
23	Supplementary information (study protocol, statistical code, model details).	Yes	Supplementary figures S1–S2 and this completed TRIPOD+AI checklist; analysis code available on request.
24	Funding and role of funders.	Yes	Funding statement: no external funding.

Item	Checklist item	Done	Location / comment
25	Conflicts of interest.	Yes	Conflicts of Interest statement: none declared.
26	Availability of data and code.	Yes	Data Availability Statement: anonymised dataset from corresponding author on reasonable request; analysis notebook available on request.
27	Ethical approval and informed consent.	Yes	IRB statement: Bioethics Committee, Zielona Góra (KEPLER 01/178/2025); informed consent obtained.

D = development; E = evaluation. Item numbering follows the TRIPOD+AI expanded checklist (version 7 February 2024).
